# The Muscles of the Eye

**Published:** 1908-12

**Authors:** 


### BOOKS AND AUTHORS.

**The Muscles of the Eye.** By **Lucien Howe,** M.A., M.D., Professor of Ophthalmology, University of Buffalo. In two vols. Vol. 2. Pathology and treatment. Illustrated. pp. 481, 8vo. G. P. Putnam's Sons. New York and London. 1908. (Cloth, $4.50).

The completion of the elaborate work of Dr. Howe on "The Muscles of the Eye," the first volume of which was reviewed in the JOURNAL in April, 1907, is an achievement which should arouse the fraternal pride of every physician who is directly or indirectly interested in ophthalmology. This volume embodies an extended study of pathology and treatment of affections of the ocular muscles, which is pursued with the same care, thoroughness and system as were shown in the first volume. Nothing of importance to the ophthalmic practitioner seems to have been omitted, and by reason of diagnostic and therapeutic bearings involved, the work has an absorbing interest, even exceeding that of the preceding volume.

Within the space at our command, it is impossible to review this work as fully as it merits. But the following outline will indicate to the reader somewhat of its scope and scientific character. It is divided into five parts, and each of these is further divided into chapters, divisions and subdivisions. Supplementing these parts there are three appendices.

Part I deals with *ocular muscle imbalance* in all of its forms as manifested in the intraocular muscles alone (accommodation), the extraocular muscles alone (heterophoria, including torsion) and in both the intra- and extraocular muscles as related to one another. The description, effects, diagnosis and treatment of the numerous varieties are presented in a most complete and practical manner, the subject occupying nearly one-half of the book, aside from the appendices. Part II embodies an extended study of *"deviations due to lesions in the extraocular muscles or in the globe (strabismus)"* and gives their definition, symptoms, diagnosis, etiology, pathology and treatment. Part III in the same manner discusses the *"deviations due to lesions in the brain or nerves (the paralyses)."* In part IV, *"atypical movements—inflammations and injuries of the muscles"* are dealt with, and in part V, the *"operations on the muscles"* are described in their whole range and variety.

In reading these pages one is deeply impressed by the amount of painstaking labor which the author has bestowed. Although he has presented many original suggestions, devices and methods, yet he has had no hobbies to ride, and has broadly and with the greatest fairness recorded the views and experiences of others, giving specific references in the text to the authorities from which he has drawn his information. The numerous illustrations are appropriate and well-executed and the literary and scientific character of the work is of high quality.

In addition to the text, proper, there are three appendices. Appendix A contains a very complete bibliography of the subjects treated, and not only indicates the laborious research and erudition of the author, but is an invaluable aid to those who may desire to pursue this theme further and refer to original authorities. The bibliographic references in this volume include numbers 825 to 2,000, grouped under headings corresponding to the various divisions of the text. The bibliography concludes with an index to the authors referred to, as is done in the first volume. Appendix B contains a continuation from the first volume of a list of questions on subjects that are suggested for further investigation. Appendix C tabulates the ophthalmological journals which may be found in certain English libraries.

The volume ends with a carefully prepared index, and portraits and sketches of a few of the ophthalmologists who have made important contributions to ocular myology.

From this outline, although much restricted, the reader may realise the amount of labor which this as well as the first volume has entailed, and the wealth of material which has here been brought together. The author is to be congratulated on the consummation of such a difficult task in such a praise-worthy manner, and the profession should cordially welcome the publication of a work having the scientific and practical value of this one.

